# Teacher stress in social interactions in the light of polyvagal theory. An ambulatory assessment approach to teachers’ heart rate and heart rate variability

**DOI:** 10.3389/fnins.2024.1499229

**Published:** 2024-12-04

**Authors:** Fabienne Kühne, Alexander Wettstein, Gabriel Jenni, Ida Schneider, Martin grosse Holtforth, Roberto La Marca

**Affiliations:** ^1^Department of Research and Development, University of Teacher Education Bern, Bern, Switzerland; ^2^Clinical Psychology and Psychotherapy, Department of Psychology, University of Bern, Bern, Switzerland; ^3^Psychosomatic Medicine, Department of Neurology, Inselspital, Bern University Hospital, Bern, Switzerland; ^4^Clinica Holistica Engiadina, Centre for Stress-Related Disorders, Susch, Switzerland; ^5^Clinical Psychology and Psychotherapy, Department of Psychology, University of Zurich, Zürich, Switzerland

**Keywords:** heart rate, heart rate variability, polyvagal theory, student aggression, teacher stress, teacher-student relationship

## Abstract

**Background:**

Teaching is a genuinely social and highly demanding task. Drawing on Porges’ phylogenetic polyvagal theory with three evolved systems and three associated behavioral responses (social engagement, mobilization, and immobilization), we investigated teachers’ heart rate and heart rate variability in social interactions using ambulatory assessments.

**Methods:**

We continuously measured heart rate and heart rate variability of 42 apparently healthy teachers on a work and leisure day with ambulatory electrocardiogram. We videotaped four consecutive, same-day lectures of each teacher. We trained observers to code student aggression and frontal teaching behaviors in an event sampling procedure with the behavior observation system for analyzing aggressive behavior in school settings. Additionally, perceived teacher-student relationship, social support from other teachers / school management, occupational complaints, and vital exhaustion were assessed by teacher self-reports.

**Results:**

Teachers showed an increased heart rate and a decreased heart rate variability on a workday compared to a leisure day, anticipatory stress before classes, as well as insufficient recovery during lunchtime. Observed student aggression and high proportion of frontal teaching were associated with lower heart rate variability, while better perceived teacher-student relationship was correlated with higher heart rate variability. Differently, teachers’ psychological strain and heart rate variability were unrelated to each other.

**Conclusion:**

Corresponding to polyvagal theory, results suggest that successful social interactions are fundamental for teachers’ favorable cardiological reactions.

## 1 Introduction

Humans are genuinely social beings and fundamentally desire to form and maintain meaningful social connections with others (Baumeister and Leary, 1995). We live in a highly specialized society that relies on cooperation and social interaction in multiple relationships. The social brain hypothesis (Dunbar, 2009) posits that relationships were a driving force behind the evolution of large brains. Accordingly, our nervous system is geared not only toward pure survival but also toward communication and interaction with other people. Whereas diverse social interactions represent powerful resources, they can also be challenging and cause stress (Umberson and Karas Montez, 2010). The present study focuses on teaching as a genuinely social profession. Teaching is characterized by high social demands, which entail a variety of stressors (Soltau and Mienert, 2010; Rothland, 2013; Nieskens, 2016). Student aggression is a strong interpersonal stressor that harms humans’ close social bonds (Baumeister and Leary, 1995; Epel et al., 2018). However, positive teacher-student relationships or support from other school professionals can also be social resources (Spilt et al., 2011; Ju et al., 2015; Aldrup et al., 2018). Drawing on the polyvagal theory, this study examines teachers’ cardiac responses in social interactions.

Teachers face multiple and complex demands (Soltau and Mienert, 2010; Rothland, 2013; Nieskens, 2016). They must adapt the curriculum to children’s individual needs, maintain discipline, monitor the class, and sanction rules (Helmke, 2009; Rothland, 2013). Above and beyond these direct classroom challenges, teachers must continuously shape interactions with colleagues, school administrators, other involved professionals, and parents (Rothland, 2013). These interactions are characterized by a high social density, simultaneity, immediacy, unpredictability, informality, and publicity (Doyle, 1986; Lortie, 2002; Herzog, 2006) and occur both verbally and nonverbally (Schilbach, 2015). Taken together, the teaching profession is characterized by high levels of stress (Aloe et al., 2014; Eurofound and EU-OSHA, 2014), with 20 to 30% of teachers reporting that their job is stressful or very stressful (Kyriacou, 2015). It can be assumed that risk factors may increase stress, while resources may reduce stress.

One of the most challenging problems for teachers in the classroom and a primary source of teachers’ stress is *student aggression* (Boyle et al., 1995; Byrne, 1999; Evers et al., 2004; Lehr, 2004; Chang and Davis, 2009; McCormick and Barnett, 2011; Dicke et al., 2014; La Marca et al., 2023; Wettstein et al., 2023a, 2023b). Generally, aggression is a strong social interpersonal stressor that harms humans’ close social bonds (Baumeister and Leary, 1995; Epel et al., 2018). Student aggression refers to any behavior students perform with the intent of causing physical or mental harm to another person who is motivated to avoid the harm (e.g., Anderson and Bushman, 2002; Wettstein, 2008). Within educational settings, student aggression can manifest in diverse ways: verbal or physical, direct (e.g., insulting, hitting), or indirect (e.g., hiding objects, spreading rumors) (Björkqvist et al., 1992).

Levels of teacher stress also vary by methodical-didactical settings employed to promote student learning. *Frontal teaching* is particularly demanding because teachers are confronted with various demands at the same time while keeping an eye on all students simultaneously (Seitz, 2007; Wettstein, 2008). Students observe their teacher from a largely inactive stance and sometimes use this to become” invisible” to the teacher and/or to progressively test his or her boundaries (Wettstein, 2008). By that, frontal teaching is more likely to be a source of stress than cooperative forms of learning or individual work.

A good *teacher-student relationship* has shown to be a key resource for teacher health (Spilt et al., 2011; Aldrup et al., 2018), as it can prevent physiological stress in teachers. La Marca et al. (2023) found that a good teacher-student relationship is associated with lower levels of hair cortisol in teachers. It also reduces disruptions such as student aggression and encourages prosocial behavior (Obsuth et al., 2017) and is positively related to students’ motivation to learn (Wentzel, 2010), academic performance (Pianta et al., 2012), as well as psychosocial development (Davis, 2003; Pianta, 2006; Wentzel, 2010; Obsuth et al., 2017).

To successfully master challenging social interactions in everyday school life, teachers also need *social support* from other teachers, school management, or administrators. Thus, support from the school team is vital and can protect against stress (Ju et al., 2015). For example, Wettstein et al. (2023a) have found evidence that support from other teachers or school administration has a protective effect on teachers’ allostatic load. In addition, social support reduces the impact of stress on blood pressure (Steptoe, 2000) and on heart rate variability (Schwerdtfeger and Schlagert, 2011; Santarpia et al., 2023).

In sum teaching is characterized by complex and potentially challenging psychosocial relationships (Doyle, 1986; Lortie, 2002; Herzog, 2006). In the classroom, it requires a good awareness of the social events and the ability to respond sensitively and appropriately to the situation. Accordingly, previous research has shown that stress severely impairs these capabilities (Bodenmann et al., 1996; Porges, 1997).

**Figure 1:**
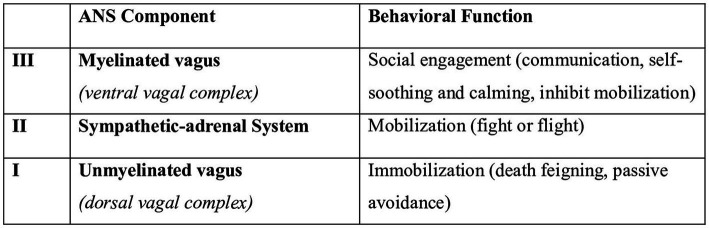
The polyvagal theory and its behavioral responses, modified from reference (Porges, 2003).

When resources to cope with environmental demands are insufficient, and the situation is perceived as threatening (Lazarus and Folkman, 1984; Friedman-Krauss et al., 2014; McCarthy et al., 2016), psychological, physiological, and behavioral stress responses may result (Lazarus, 1991; Mauss and Robinson, 2009). The *polyvagal theory* (Porges, 1995, 2021) is a phylogenetic model that specifies the association between the evolution of the autonomic nervous system (ANS) and social communication (affective experiences, emotional expression, vocal communication, facial gestures, and social behavior), which is central for the teaching profession. The ANS comprises the sympathetic nervous system (SNS, usually associated with activation and energy mobilization) and the parasympathetic nervous system (PNS, responsible for vegetative and restorative functions) (Ehlert et al., 2013). The polyvagal theory proposes that the PNS is additionally divided into two distinct branches: the ventral and the dorsal vagus (Porges, 1995). The theory also states that the ANS has developed during evolution (Porges, 1995), and the different phylogenetically old structures are supposedly associated with distinct behavioral strategies [i.e., social engagement, mobilization (fight or flight), and immobilization; Porges (1997, 2001)] (Figure 1).

### 1.1 Social engagement

A person confronted with social challenges usually tries to overcome them through social interaction (Porges, 2001). The ventral vagus, being the phylogenetically youngest part of the ANS, is considered responsible for this. It is only found in mammals such as humans and forms the basis for social communication (Porges, 1995, 1997). The functional neural organization is called the social engagement system (Porges, 2001, 2003). The ventral vagus is myelinated, which enables rapid transmission of nerve signals and, thus, highly dynamic adaptation to the environment (Porges, 1995). It inhibits sympathetically controlled mobilization (i.e., fight or flight-behavior), regulates the heart rate, and acts as a vagal brake – preventing the sympathetic nervous system from influencing the heart (Porges, 1997). Central to activating the social engagement system is the perception of safety about the internal or external situation (Porges, 2003, 2022).

### 1.2 Mobilization (fight or flight)

When a challenge is not resolved through social interaction or when a situation is subjectively perceived as physically or socially threatening, the sympathetic nervous system is assumed to be activated (Porges, 2003). The sympathetic nervous system is the second oldest part of the ANS (Porges, 1997) and prepares the organism for the mobilization response (Porges, 1997). Physiologically, its activity is associated with the body’s energy-and oxygen provision needed to fuel a rapid response to danger (Porges, 1997). However, this mode reduces effective social communication and interaction skills (Porges, 1997). In service of facilitating fight (emotion of anger) or flight (emotion of fear) with a quickening of thoughts and scanning attention for potential sources of threat (Porges, 1997).

### 1.3 Immobilization

If mobilization is insufficient to deal with the stressor, the dorsal vagal complex is assumed to come into play (Porges, 1995), which manifests in immobilization behavior (e.g., decreasing heart rate, decline of bloody oxygen, sense of dread, and solidification of the body) and may perceived as “playing dead.”

Assessing the level of safety in the environment is critical for the individual to determine which behavioral strategies (social engagement, mobilization, immobilization) will be activated (Porges, 2003). The polyvagal theory proposes *neuroception* as a subconscious neural process that assesses the environment’s safety, danger, or life-threatening nature (Porges, 2003). The ANS is also assumed to be organized hierarchically, i.e., the social engagement system is mobilized first when safety is perceived (Porges, 1995). If this is insufficient or the situation is assessed as dangerous or even life-threatening, phylogenetically older parts of the ANS are activated (Porges, 2003). In a healthy organism, the SNS and PNS activity is in dynamic relative balance (Shaffer et al., 2014).

### 1.4 Stress and ANS

Widely used measures of ANS activity are heart rate (HR) and heart rate variability (HRV), as cardiac activity is largely under the control of ANS processes (Task Force of the European Society of Cardiology the North American Society of Pacing and Electrophysiology, 1996; Thayer et al., 2009; Shaffer and Ginsberg, 2017). The heart is under tonic inhibitory control of the vagus nerve, which functions as a vagal brake (Levy, 1990; Uijtdehaage and Thayer, 2000).

*HR* corresponds to the number of contractions (beats) per minute (Pinel, 2010). *HRV* refers to the beat-to-beat alterations between successive heartbeats and reflects the balance of the ANS (Task Force of the European Society of Cardiology the North American Society of Pacing and Electrophysiology, 1996). There are many different HRV parameters. RMSSD (root mean square of successive differences between normal heartbeats) and SDNN (Standard Deviation of the NN Interval) are particularly often used in research (Shaffer et al., 2014). *RMSSD* corresponds to the beat-to-beat variance in heart rate and is considered the standard measure of parasympathetic (vagally) mediated cardiac regulation (Shaffer et al., 2014). *SDNN* expresses the overall variability of the temporal fluctuations of the heartbeat (Shaffer et al., 2014). The SDNN indicates the ANS’s regulatory capacity and the cardiovascular system’s adaptability (Shaffer et al., 2014).

Under stress, there is usually an increase in HR and a decrease in vagally-related HRV indices (Task Force of the European Society of Cardiology the North American Society of Pacing and Electrophysiology, 1996). Elevated HRV is associated with a better ability to regulate stress and arousal (Thayer and Lane, 2000, 2009) and indicates a good self-regulatory capacity (emotion regulation) and adaptability (McGee et al., 2023). More work stress is usually associated with decreased HRV – an indicator of reduced vagal activity (Chandola et al., 2010).

The relationship between stress and cardiac parameters is well established (e.g., Allen et al., 2014), but there have been few studies examining these in the context of concrete stressful situations in teachers’ everyday lives (Beuchel and Cramer, 2023). A recent review indicates generally inconsistent results on the relationship between stress and cardiovascular parameters in teachers (McGee et al., 2023). Specific studies found that teachers have a higher HR (Schwerdtfeger et al., 2008) or lower HRV (Wettstein et al., 2020) on workdays compared to leisure days. In addition, a higher HR among teachers was also observed in teaching compared to organizational work (Scheuch and Knothe, 1997). A recent ambulatory assessment study found no significant correlations between perceived job demands or resources and HRV (Schmid and Thomas, 2020).

### 1.5 Anticipatory stress and recovery

Pre-lesson phases are particularly stressful for teachers. Scheuch and Knothe (1997) found a strong effect of anticipatory stress in teachers with particularly high heart rates before classes. Pieper et al. (2007) have found elevated HR and decreased HRV in teachers during worry episodes and stressful events compared to neutral periods. Anticipatory stress in the form of work-related worries had a particularly strong effect on HR and HRV in teachers.

*Recovery.* Some studies have investigated the physiological recovery of teachers after lessons. Steptoe (2000) has shown a lower HR in the evening compared to working hours. In another study Steptoe et al. (1999) found a reduction in cardiac parameters during leisure time compared to working time only in teachers who reported low levels of work stress. Teachers who reported high work stress failed to recover sufficiently from work stress in their leisure time (Steptoe et al., 1999). In another study, physiological relaxation in teachers, as measured by high-frequency power (HRV frequency domain parameter), tended to occur only during holidays but not weekends (Ritvanen et al., 2004).

### 1.6 Acute and chronic stress

Although in the teaching profession, the connection between stress and cardiovascular parameters has been increasingly demonstrated, some studies have failed to find an association between stressful events and changes in HR or HRV in teachers (e.g., Pieper et al., 2010).

The acute stress response is regarded as an adaptive adjustment of the organism to internal or external challenges (McEwen, 2007). Repeated or prolonged exposure to stress can harm the body (Porges, 2003), and in the long term, chronic stress can contribute to the development of psychological, psychosomatic, and physiological illnesses (e.g., depression, concentration problems, sleep disorders, and altered ANS activity) (Kokkinos, 2007; McEwen, 2007).

One stress-related outcome is *psychological strain.* Psychological strain can be operationalized by vital exhaustion (Appels and Mulder, 1989) or occupational problems (Hagemann and Geuenich, 2009). *Vital exhaustion* is a psychosomatic state characterized by excessive fatigue, lack of energy, increased irritability, and feelings of demoralization (Appels and Mulder, 1989). *Occupational problems* include negative feelings related to work (Hagemann and Geuenich, 2009).

Chronic stress has been shown to jeopardize health and well-being, reduce teaching quality, and harm student motivation and performance (Klusmann et al., 2016). In the long run, stress can impair teachers’ health, potentially leading to early dropouts from the profession (Ingersoll, 2001; Friedman, 2006) and may be associated with high subsequent health costs (Künzi and Oesch, 2016).

### 1.7 The present study

Research on teacher stress is mainly based on questionnaires for measuring subjective stress experiences, ignoring essential data on real-time physiological stress reactions (Krause et al., 2013; Wettstein et al., 2020). In self-reports, teachers have almost unanimously identified challenges in social interactions as the main stress factor (Scherzinger and Wettstein, 2019). However, it is still unclear under which conditions challenging interactions trigger acute psychological and physiological stress reactions in teachers (Wettstein et al., 2020). Only a few studies have investigated cardiovascular parameters in everyday lives of teachers (Pieper et al., 2007, 2010; Schmid and Thomas, 2020; Beuchel and Cramer, 2023). To the best of our knowledge, no studies have associated cardiac parameters in teachers with video-taped, observer-rated student aggression. Going beyond previous studies (Pieper et al., 2007, 2010; Schmid and Thomas, 2020; Beuchel and Cramer, 2023), we also examine the association between perceived teacher-student relationship or the methodical-didactical setting and cardiac parameters. Also, teachers’ diurnal courses of HR and HRV on workdays and leisure days have been insufficiently investigated to date. In the present study, we aim to deepen our knowledge about teachers’ cardiac responses to potentially stressful interactions and set out to identify potential risk factors and resources of the teaching profession. We will base our research on polyvagal theory and analyze teacher stress in the context of social interactions.

The present study set out to investigate differences in HR and HRV between workdays and leisure days and to examine whether there are anticipatory stress reactions on a workday. Additionally, we aimed to examine how potential risk factors (student aggression, methodical-didactical setting) and resources (perceived teacher-student relationship, perceived social support) are associated with teachers’ HR and HRV in everyday working life. We are also interested in the relationship between cardiac parameters and psychological strain as a stress-related consequence.

Going beyond previous studies on teacher stress, we included self-reports and used ambulatory assessment methods in the field, including observational and physiological measures, to research the participants in their natural habitats (Trull and Ebner-Priemer, 2013). This enables an ecologically valid assessment of teacher stress in everyday work and helps us better understand the interplay between psychological, physiological, and social factors.

We tested the following hypotheses:

*H1:* Compared to leisure days HR will be higher and HRV will be lower on workdays (hypothesis 1) (Chandola et al., 2010; Steptoe and Kivimäki, 2012; Järvelin-Pasanen et al., 2018).

*H2:* HR will be higher and HRV lower before classes than during classes (hypothesis 2) (Porges, 1997; Pieper et al., 2007).

*H3:* Assumed risk factors (observed student aggression and frontal teaching) will be related negatively and resources (perceived positive teacher-student relationship and perceived support within the school team) positively with teachers’ HRV (hypothesis 3). Inverse associations are expected for HR (Porges, 2003; Aloe et al., 2014; Dicke et al., 2014).

*H4:* Psychological strain variables (vital exhaustion and occupational problems) will be positively associated with HR and negatively associated with HRV (hypothesis 4) (Thayer and Lane, 2009).

## 2 Materials and methods

### 2.1 Participants

Teachers were recruited via flyers and circular emails sent to school administrations in the canton of Bern (Switzerland). The final sample consisted of 42 apparently healthy teachers (28 females; *M*_age_ = 39.66, *SD* = 11.99, range = 23–63) teaching at least 16 weekly lessons.

Participants came from 39 schools within the canton of Bern and taught grade levels ranging from kindergarten and elementary school (1st to 6th grade; *n* = 27) to secondary school (7th, 8th, and 9th grade; *n* = 12) and to high school and vocational school (10th, 11th, and 12th grade; *n* = 3). On average, teachers had 13.35 years of teaching experience (*SD* = 11.07, range = 1–40). We included different school types and levels to capture a comprehensive picture of the stress factors and resources in the entire education sector.

Exclusion criteria besides working outside of the canton of Bern were smoking more than 10 cigarettes per day, pregnancy, drug or substance abuse, use of cardiovascular drugs or other medication in the past 2 months (besides herbal medicine), consumption of psychoactive substances in the last 4 weeks, more than two standard alcoholic drinks per day, long-haul flights in the previous 2 weeks, acute infections, and cardiovascular or other chronic diseases. The school principals were informed, and all participating teachers signed informed consent.

### 2.2 Design and procedure

The present study is part of a larger, longitudinal ambulatory assessment project examining psychobiological stress in teachers (Wettstein et al., 2021, 2023a, 2023b; La Marca et al., 2023). As part of a multimodal approach, it comprises self-report, observational, and physiological measures to increase the ecological validity and to reduce potential common method bias.

A brief telephone interview was conducted to ensure that teachers met the inclusion and exclusion criteria. Teachers’ physiological stress responses were collected continuously through electrocardiogram (ECG) from awaking until 7:00 pm throughout one workday and one leisure day (the order of the days was counterbalanced). The teachers also kept diaries to assess their activities on each measurement day.

The study was approved by the Ethics Committee of the Canton of Bern and the Internal Review Board (IRB) of the University of Bern. It was conducted strictly in accord with current national data protection laws and with the declaration of Helsinki.

### 2.3 Measures

In advance of the study, teachers provided information on demographic variables (sex, age, years of teaching experience, and number of lessons taught in the observed class). In addition, they completed paper-pencil questionnaires on stress and resources in the teaching profession (i.e., perceived teacher-student relationship, social support from other teachers and the school management, teachers’ occupational problems, and vital exhaustion). The corresponding questionnaires were sent to them.

At an initial appointment at the PHBern, weight and height were recorded to determine the BMI. BMI was calculated by dividing the teachers’ weight (kg) (Seca 813; Reinach, Switzerland) by the square of their height (m) (Seca 213; Reinach, Switzerland). The procedure and the use of the ECG sensor were explained and practiced.

#### 2.3.1 Behavioral observation

We equipped each classroom with a GoPro camera with an integrated wide-angle function and a dictaphone to record the methodological-didactical setting and student aggression. The camera was fixed in the center of the classroom on the back wall at a height of 180 cm. We obtained informed consent from the parents of children in the classrooms. Filming took place during four consecutive lessons on one workday. We synchronized the classroom videos with the ECG sensor. Two independent observers coded the video recordings using an event sampling procedure (Faßnacht, 1995) based on the behavior observation system for analyzing aggressive behavior in school settings BASYS (Wettstein, 2008). The focus was on the methodological-didactic setting and student aggression. In the present study, we focused on frontal teaching. An additional observer coded 10% of the video material to assess observer agreement. This resulted in an observer agreement of 0.80 for student aggression and 0.92 for classroom settings (weighted Cohen’s Kappa, correcting for the possibility of the agreement occurring by chance). Video coding was performed using MAXQDA Analytics Pro 2020 version 20.4.1 (VERBI Software, 2019). The mean value for the number of student aggressions and the proportion of frontal teaching was calculated for each teacher.

#### 2.3.2 Psychometric measures

The perceived teacher-student relationship was measured with a subscale of the Classroom Questionnaire (Wettstein et al., 2016). Six items were rated on Likert scales ranging from 1 (not true) to 4 (true). A sample item is “I have a good relationship with my students.” Reliability was Cronbach’s *α* = 0.73.

Two items assessed perceived social support from other teachers (“If I need advice, other teachers are there for me.”) and the school management (“If I feel overwhelmed, I am supported by the school management.”), using 5-point Likert-scales ranging from 1 (strongly disagree) to 5 (strongly agree). The correlation between the two items was moderate, with a Spearman-Brown coefficient of *r_tt_* = 0.54. The two items were, therefore, analyzed as individual items.

*Teachers’ occupational problems* were assessed with a subscale of the Burnout Screening Scales (BOSS I; Hagemann and Geuenich, 2009). Ten items were rated on Likert scales ranging from 1 (does not apply) to 6 (applies strongly). A sample item is “I have lost the joy of my work.” Reliability was Cronbach’s *α* = 0.91.

*Vital exhaustion* was assessed using the German translation (cf. Rau et al., 2010) of the Maastricht Vital Exhaustion Questionnaire (MQ; Appels et al., 1987). The scale consists of 21 items assessing fatigue, difficulties falling asleep, apathy, irritability, energy loss, depression, and waking up exhausted. A sample item is: “Do you sometimes feel as if your body is like a battery that is losing its power?.” The 21 items could be rated on a three-point scale, from “statement is not true” (1) to “undecided” (2) or “true” (3). The scores of each item were added to calculate the total score, with higher scores indicating increased vital exhaustion. The reliability was Cronbach’s α = 0.88.

#### 2.3.3 Electrophysiological measures

In order to take methodological aspects in HRV measures sufficiently into account (Laborde et al., 2017), we followed the guidelines of the Task Force of the European Society of Cardiology the North American Society of Pacing and Electrophysiology (1996). We recorded the ECG with the EcgMove4 sensor from Movisens Version 1.9.31.0 (movisens GmbH, Karlsruhe, Germany) with adhesive electrodes that are easy to apply. It records a single-channel ECG at a sampling rate of 1,024 Hz. Upon waking, the teachers applied the sensor using adhesive electrodes, and ECG data were recorded continuously until 7:00 pm.

The processing and analysis of the ECG data were conducted using Kubios HRV Premium version 3.5.0 (University of Eastern Finland, Kuopio/Finland; Tarvainen et al., 2014). Since artifacts significantly affect the reliability of HRV measurements (Tarvainen et al., 2014), raw data was edited manually in Kubios to review peak detection and ensure proper removal of artifacts (e.g., missing, extra, or misaligned beats) and ectopic beats. We followed Tarvainen et al. (2014) guidelines regarding artifact correction. To prevent a significant bias in the analysis results, the number of artifacts, even if corrected, did not exceed 5%.

To determine differences in cardiac parameters between the workday and the leisure day (H1), HR and HRV values were averaged on both days from waking until 7:00 pm. In addition, on workdays, we compared the cardiac parameters in the hour before the lesson begins (the time the teacher spends alone in the classroom before the lesson starts) with the value during the following lesson (i.e., 7–8 with 8–9) to check whether there were anticipatory stress reactions (H2). The averaged HR and HRV values on the work and leisure days were also used to test hypotheses 3 and 4.

After artifact correction (Catalano, 2002), HR, RMSSD, and SDNN were exported into SPSS and analyzed using a within-subject design (Laborde et al., 2017).

### 2.4 Data analysis

The Shapiro–Wilk test was used for each variable to test normal distribution. Due to non-normal distribution, the following variables were log-transformed before use for calculations: RMSSD and SDNN on work and leisure days, observed direct and indirect student aggression, frontal teaching, perceived teacher-student relationship, support from other teachers and the school management, occupational problems, vital exhaustion, BMI, and age. When correlated significantly (partial correlations), control variables (age, sex, and BMI) were considered. In addition, correlations between ECG and questionnaire variables were controlled for time differences between the two measurements (delay). We have calculated whether there are differential effects at the different school levels. No effects were found. Additionally, RMSSD on work and leisure days was controlled for age and BMI. SDNN on a workday was controlled for age, and, on a leisure day, for BMI. HR at work and leisure days were controlled for age and sex.

All analyses were conducted using IBM SPSS Statistics (Version 29). To compare the work with the leisure day (H1), we conducted a one-way repeated measures MANOVA with day as a factor (workday and leisure day), HR, RMSSD, and SDNN as dependent variables, and sex, age, and BMI as covariates. Regarding concerns about the non-normal distribution of RMSSD and SDNN, we used the log-transformed scales, and the Box test for equality of covariance matrices was not significant. For pairwise comparisons between times (H2), no repeated measures ANOVAs (HR) or Friedman tests (RMSSD and SDNN) were calculated, as there were missing values and only specific comparisons were of interest. Instead, single paired t-tests (HR) and Wilcoxon tests (RMSSD and SDNN) were calculated. The resulting *p*-values were adjusted with the false discovery rate control (Glickman et al., 2014). Descriptive statistics and bivariate correlations were computed for all variables to investigate hypotheses H3 and H4.

## 3 Results

### 3.1 Workday and leisure day

A one-way repeated measures MANOVA resulted in a significant effect of day [*F*(28,3) = 3.33, *p* = 0.34, partial *η*^2^ = 0.26, Wilks-Lambda = 0.74, *n* = 34] when controlling for sex, age, and BMI. The partial η^2^ of 0.26 indicates a large effect (Cohen, 1988). Bonferroni corrected pairwise comparisons revealed that HR was significantly (*p* = 0.034) higher on the workday (*M* = 78.81, *SD* = 7.11) than on the leisure day (*M* = 76.16, *SD* = 9.25). RMSSD values were significantly lower (*p* < 0.001) on the workday (*M* = 34.08, *SD* = 15.17) compared to the leisure day (*M* = 38.84, *SD* = 20.90). SDNN was also significantly (*p* = 0.028) lower on the workday (*M* = 80.49, *SD* = 46.97) than on the leisure day (*M* = 98.28, *SD* = 59.68).

Particularly, RMSSD was significantly lower on the workday compared to the leisure day at 1 pm (*Mdn_work_* = 22.68, *Mdn_leisure_* = 33.59, *z* = −3.03, *p_adj_* = 0.027, *n* = 32, *r* = 0.54, strong effect) and 2 pm (*Mdn_work_* = 27.01, *Mdn_leisure_* = 29.41, *z* = −2.84, *p_adj_* = 0.027, *n* = 32, *r* = 0.50, strong effect). For HR and SDNN, no significant difference was found between the same time on the workday and leisure day.

### 3.2 Anticipatory stress and diurnal course

**Figure 2:**
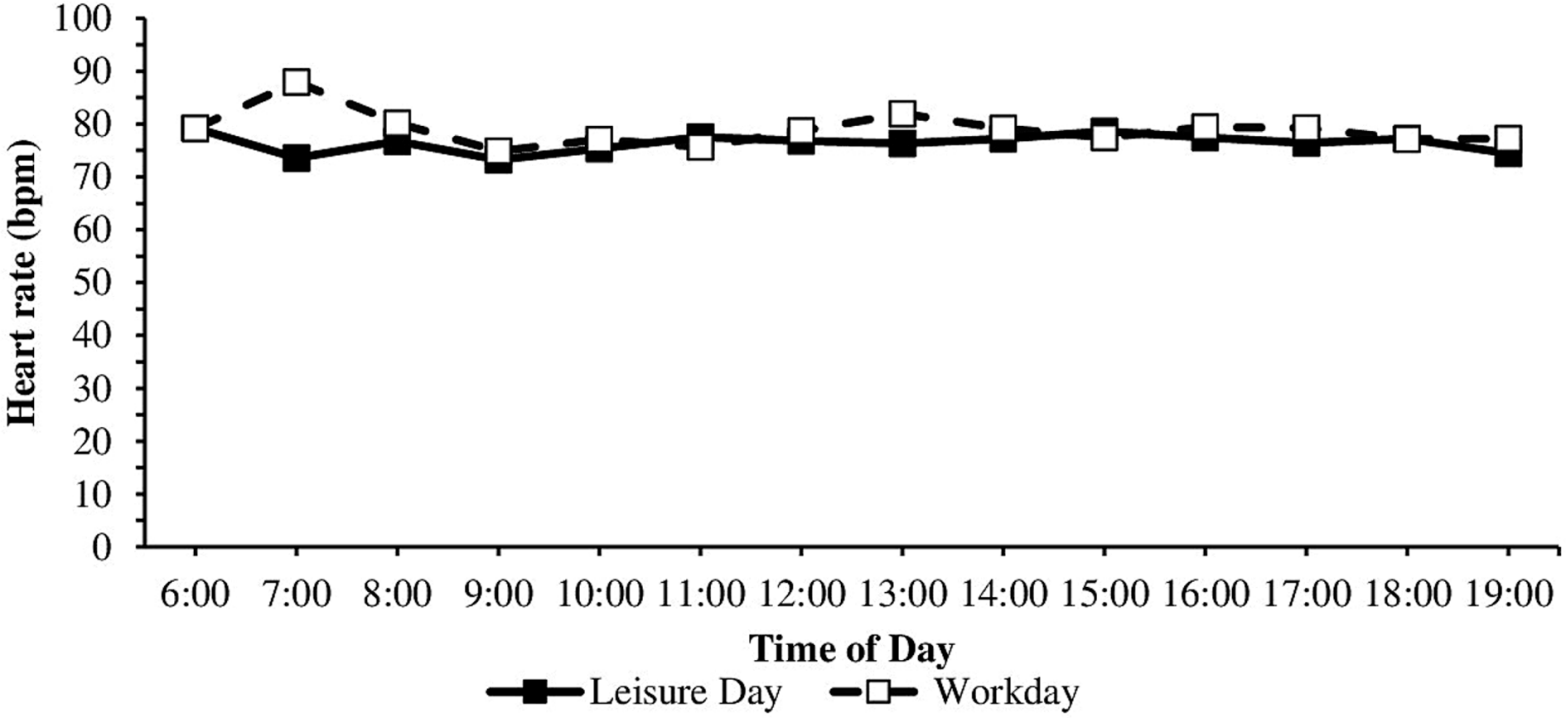
HR during the workday and leisure day between 6 am and 7 pm. *n* workday = 28–38, *n* leisure day = 5–38.

**Figure 3:**
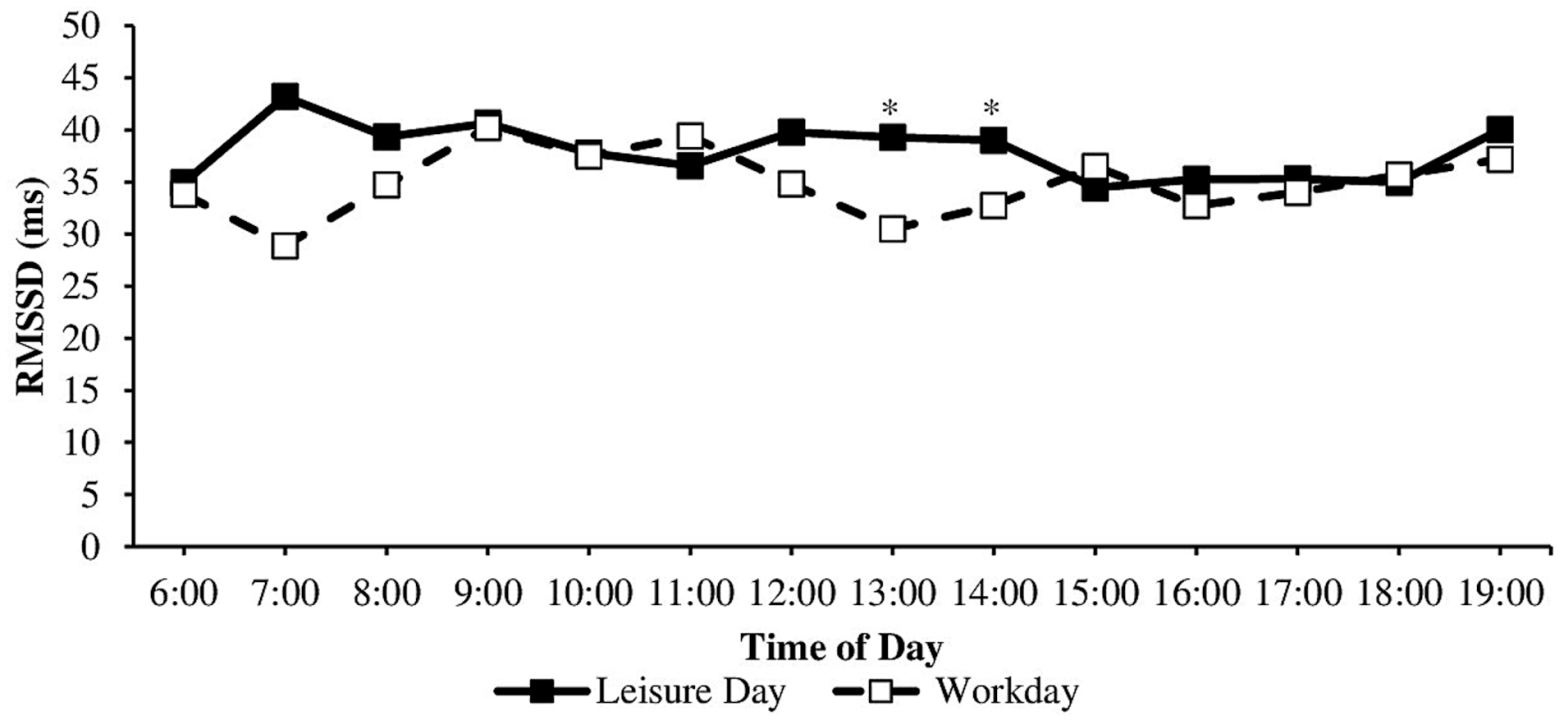
RMSSD during the workday and leisure day between 6 am and 7 pm. *n* workday = 28–38, *n* leisure day = 5–38, RMSSD, root mean square of successive differences in milliseconds, *indicating significant (*p_adj_* < 0.05) differences between work and leisure day.

**Figure 4:**
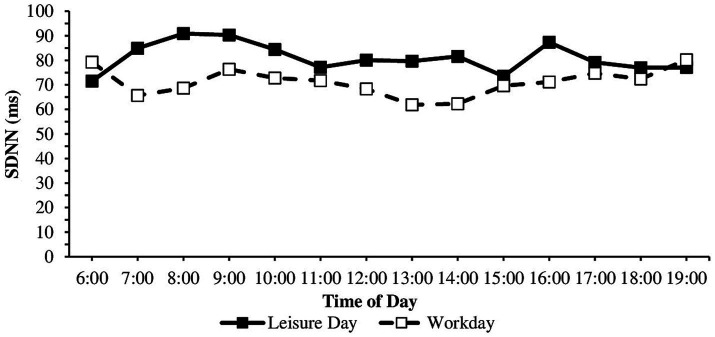
SDNN during the workday and leisure day between 6 am and 7 pm. *n* workday = 28–38, *n* leisure day = 5–38, SDNN, standard deviation of the NN interval in milliseconds.

Regarding anticipatory stress, HR was higher, and RMSSD and SDNN were lower before than during classes (Table 1). The course of cardiac parameters throughout a workday was investigated further in an exploratory manner. Dependent sample t-tests showed significant differences in mean HR on workdays between 6 and 7 am, 7 and 8 am, 8 and 9 am, 11 and 12 am, and 12 am and 1 pm. Wilcoxon tests showed that RMSSD values significantly differed on workdays between 6 and 7 am, 7 and 8 am, 8 and 9 am, 11 and 12 am, 12 am and 1 pm, and 2 and 3 pm. SDNN values significantly differed on workdays between 6 and 7 am, 8 and 9 am, 12 am and 1 pm, and 2 pm and 3 pm (Table 1). For the direction of the effects (higher-lower), consult the means and medians in Table 1 and the diurnal courses in Figures 2–4. HR was higher, and RMSSD was lower at 1 pm than 12 am. There were no significant differences on the leisure day between any two times for HR, RMSSD, and SDNN (all *p* > 0.05).

**Table 1:** Significant differences between the times within the workday for HR, RMSSD and SDNN.

Variable	Time_1_	Time_2_	M_Time1_	M_Time2_	*t*	*p_adj_*	*n*	*r*
HR	6 am	7 am	79.14	86.12	−3.37	0.010	28	0.54
	7 am	8 am	87.85	80.16	5.71	< 0.001	34	0.71
	8 am	9 am	79.48	74.96	6.23	< 0.001	35	0.73
	11 am	12 am	75.72	78.40	−2.56	0.041	35	0.40
	12 am	1 pm	78.65	81.84	−2.54	0.041	36	0.39
			***Mdn***_**Time1**_	***Mdn***_**Time2**_	***z***			
RMSSD	6 am	7 am	27.67	23.73	−2.35	0.041	28	0.44
	7 am	8 am	23.73	29.26	−3.68	0.002	34	0.63
	8 am	9 am	30.13	33.24	−4.41	< 0.001	35	0.75
	11 am	12 am	32.01	28.71	−2.47	0.035	35	0.42
	12 am	1 pm	28.51	22.68	−3.06	0.010	36	0.51
	2 pm	3 pm	27.69	27.08	−2.77	0.018	37	0.46
SDNN	6 am	7 am	45.93	46.27	−3.07	0.007	28	0.58
	8 am	9 am	75.24	71.02	−3.55	0.004	35	0.60
	12 am	1 pm	51.48	57.07	−3.46	0.004	36	0.58
	2 pm	3 pm	61.68	72.36	−3.07	0.007	37	0.51

*n* = 28–37, RMSSD, root mean square of successive differences, SDNN, standard deviation of the NN Intervall, HR, heart rate, *M*, mean, Mdn, median, *p*_adj_ = false discovery rate control adjusted *p*, *r* = Pearson’s *r* effect size (*r* > = 0.10 weak effect, *r* > = 0.30 medium effect, *r* > = 0.50 strong effect; Cohen, 1988), two-tailed.

### 3.3 Risk factors and resources

Table 2 present means, standard deviations, and bivariate Pearson correlations between HR, RMSSD, and SDNN at work and leisure days, observed direct and indirect student aggression, frontal teaching, perceived teacher-student relationship, support from other teachers and the school management, and control variables.

**Table 2:** Descriptive statistics and intercorrelations.

Variable	HR work	HR leisure	RMSSD work	RMSSD leisure	SDNN work	SDNN leisure
Observed direct student aggression	0.46**	0.22	−0.36*	−0.27	−0.25	−0.17
Observed indirect student aggression	0.30*	0.11	−0.25	−0.18	−0.13	−0.01
Frontal teaching	0.21	0.41**	−0.24	−0.29*	−0.32*	−0.20
Perceived teacher-student relationship	0.07	−0.01	0.31*	0.13	0.25	0.44**
Support from other teachers	−0.08	−0.28	0.24	0.22	0.04	0.14
Support from the school management	0.07	0.17	0.01	0.20	−0.10	0.09
Occupational problems	−0.21	−0.16	−0.07	0.00	0.07	0.20
Vital exhaustion	−0.22	−0.02	0.23	0.08	0.29*	0.40**
BMI	0.07	0.27	−0.28*	−0.46**	−0.17	−0.27*
Age	−0.44**	−0.38*	−0.38**	−0.40**	−0.40**	−0.21
Sex^a^	−0.54**	−0.48**	−0.04	−0.07	−0.15	−0.18
*M*	78.68	75.76	36.32	38.61	81.08	99.20
*SD*	6.81	9.06	18.21	20.05	47.27	58.49

*N* = 42, workday: *n* = 39, leisure day: *n* = 37, RMSSD, root mean square of successive differences, SDNN = standard deviation of NN intervals, HR, heart rate, BMI, body mass index. a Sex: 0 = female, 1 = male. *p < 0.05, **p < 0.01, one-tailed.

RMSSD and SDNN (workday: *r* = 0.47, *p* = 0.002; leisure day: *r* = 0.37, *p* = 0.015) as well as RMSSD and HR were weakly to moderately associated (workday: *r* = −0.23, *p* = 0.090; leisure day: *r* = 0.47, *p* = 0.003). SDNN and HR were not significantly associated (workday: *r* = −0.02, *p* = 0.454; leisure day: *r* = −0.03, *p* = 0.442).

On workdays higher levels of direct student aggression were associated with higher HR and lower RMSSD, and higher levels of observed indirect student aggression were positively associated with HR. Teachers practicing more frontal teaching in their classes had lower RMSSD on leisure days, lower SDNN on workdays and higher HR on leisure days. While high RMSSD on workdays and high SDNN on leisure days were related to a perceived good teacher-student relationship, support from other teachers and the school management was not related to HR, RMSSD and SDNN on workdays.

### 3.4 Psychological strain

There were hardly any associations between psychological strain variables and cardiac measures. Occupational problems were not associated with teachers’ HR and HRV (Table 2). Surprisingly, there was a positive association between vital exhaustion and SDNN on both work and leisure days.

## 4 Discussion

The present study aimed to investigate the course of cardiac responses (HR and HRV) over a teacher’s workday and to clarify whether these differ in comparison to a leisure day using ambulatory assessment methods. We focused on risk factors and resources potentially increasing or reducing stress in the teaching profession, on occupational problems, and on vital exhaustion.

Teachers had higher HR and lower HRV levels on workdays than on leisure days. On workdays, teachers showed anticipatory stress (higher HR and lower HRV) before classes and an insufficient recovery at lunchtime. Also on workdays, both higher HR and lower HRV were associated with observer-rated direct student aggression. While a higher amount of frontal teaching was associated with lower HRV, a good perceived teacher-student relationship was associated with more favorable cardiac responses, i.e., higher levels of HRV. Other teachers’ and the school management’s support was unrelated to HR and HRV on workdays, and there was hardly any association between psychological strain variables and cardiac measures. Surprisingly, *high* levels of SDNN were associated with vital exhaustion on both work and leisure days.

### 4.1 Work and leisure day

As hypothesized, teachers showed an elevated HR and a reduced HRV on workdays compared to leisure days (hypothesis 1). This result aligns with previous studies showing that more work stress is associated with increased HR (Steptoe and Kivimäki, 2012) and reduced HRV (Chandola et al., 2010; Järvelin-Pasanen et al., 2018). Considering polyvagal theory (Porges, 1995, 1997, 2001), higher HR and lower HRV on workdays compared to leisure days speak for a state of increased physiological arousal during teaching. This can be explained using two different approaches. On the one hand, it may reflect a transitory removal of the vagal brake (reduced vagal tone), enabling a rapid increase in cardiac output without activating the sympathetic-adrenal system (Porges, 1997). Elevated HR and low HRV, on the other hand, may also indicate an activation of the sympathetic nervous system, which prepares the organism for mobilization through augmented energy levels and optimized cognitive readiness. Increased sympathetic nervous system activation is found particularly in prolonged stressful situations, when the environment is not perceived as safe, or when a situation cannot be resolved through social interaction (Porges, 1997, 2003; Kreibig, 2010). These conditions are usually found in the classroom, which is particularly demanding due to the simultaneity of the many and complex challenges such as classroom management, student interactions, and administrative work (Soltau and Mienert, 2010; Rothland, 2013; Nieskens, 2016).

Our results are partially consistent with previous findings demonstrating higher HR (Schwerdtfeger et al., 2008) or lower HRV (Wettstein et al., 2020) during work compared to leisure time in teachers. In contrast to Schwerdtfeger et al. (2008), Wettstein et al. (2020) found no differences in HR when comparing a workday and a leisure day of the same individual. As the latter study is a pilot study, it included a relatively small sample, which may not have had sufficient statistical power to detect smaller differences.

Reduced HR and increased HRV on leisure days likely indicate that teachers’ organisms perceive these days as low-threat environments, allowing for downregulating stress responses and promoting health, growth, and restoration (Porges, 1995). This is achieved by increasing the influence of the vagal brake on the cardiac pacemaker, which slows down the heart and inhibits the mobilization response of the sympathetic nervous system (Porges, 1997). The abovementioned finding aligns with the theory’s premise that the ANS is finely attuned to safety and danger cues within the social context (Porges, 2003).

Overall, the teachers showed a lower HR and a higher HRV on the leisure days than on the working days. However, when analyzing the teachers at the individual level, some of the teachers had slightly lower RMSSD and SDNN values on their leisure days than on their working days. It can be assumed that stressors also occur in private life, which can influence physiological values. Different physical activity levels on working and leisure days (e.g., housework like cleaning) or moderate exercise (cycling, walking) could also have influenced the cardiac parameters. However, teachers were instructed not to engage in excessive sport the day before and on the examination day.

### 4.2 Anticipatory stress and diurnal course

As expected in hypothesis 2, teachers showed a heightened physiological arousal (higher HR and lower HRV) before class. This finding aligns with an earlier study by Monat et al. (1972) finding that teachers’ anticipation of classroom interactions triggers stress responses. The elevation of HR may signify a shift toward a mobilized state, potentially linked to the body’s preparation for anticipated socially demanding situations (Porges, 1997). In light of the transactional stress model (Lazarus and Folkman, 1984), the anticipatory stress before lessons can be viewed as a product of primary and secondary cognitive appraisals, by which teachers evaluate potential demands of upcoming social interactions and their ability to cope with them.

In line with theory, Pieper et al. (2007) found that anticipatory stress-related worry episodes were associated with increased HR and decreased HRV in teachers, with particularly strong effects observed for work-related concerns. Similarly, Scheuch and Knothe (1997) observed particularly high HR values before lessons as an anticipatory stress reaction.

Changes in cardiac parameters throughout a workday were investigated in an exploratory manner. There were higher HR and lower HRV at midday on workdays compared to the lesson before, which may indicate that lunchtime is particularly stressful for teachers and does not provide sufficient recovery. Instead of relaxing, teachers may use lunchtime on workdays as efficiently as possible by completing organizational tasks (e.g., copying teaching materials and answering emails) or preparing lessons. Teacher colleagues commonly interact during lunchtime, discussing various school-related topics such as organizational matters and individual student behavior. The higher HR and lower HRV values may also indicate anticipatory stress effects when teachers are cognitively dealing with the upcoming challenges of teaching.

### 4.3 Risk factors and resources

We expected that risk factors (student aggression, frontal teaching) are positively associated with HR and negatively with HRV in teachers, whereas resources (perceived teacher-student relationship and perceived support from the school team) show the opposite effect. Hypothesis 3 was only partially confirmed. Observed student aggression is related to higher HR and lower HRV (RMSSD). Teachers practicing more frontal teaching showed lower HRV (SDNN) values. Teachers who perceived a better relationship with their students showed higher HRV values (RMSSD) but no change in the HR values. Contrary to expectations, there was no significant association between self-rated perceived social support and HR or HRV.

Aggressive student behavior represents a major source of teacher stress (Aloe et al., 2014; Dicke et al., 2014). It elicits physiological stress reactions within teachers, characterized by heightened HR and reduced HRV. Physiologically, these responses manifest as an augmentation in sympathetic arousal and a diminished parasympathetic influence. Based on earlier observational studies on the detection rate of student aggression by teachers (Wettstein, 2008), it must be assumed that teachers are not consciously aware of a considerable proportion of aggression due to the pressure to act in class and the complexity of the social situation. Considering the polyvagal theory, neuroception as a subconscious neural process (Porges, 2003) might be responsible for the fact that teachers still react physiologically to these incidents.

Neurophysiological states promoting mobilization behavior tend to be incompatible with social engagement behaviors (Porges, 2001). As a result, teachers seem to be less able to deal flexibly with challenging student behavior via communication in these moments. Moreover in stressful situations, their repertoire of coping strategies is likely restricted and rigidified, limiting their ability to respond adaptively to student aggression (Porges, 1995; Thayer and Lane, 2000). Teachers with a lower HRV may also be less successful in regulating their emotions and are, therefore, less likely to show cooperative behavior (Lischke et al., 2018). Consequently, there is a risk that challenging classroom situations will persist or exacerbate.

A perceived positive teacher-student relationship can have a protective effect and is likely associated with enhanced vagal tone and, consequently, with higher HRV (Porges, 2001, 2003). Through the subconscious process of neuroception, a perceived positive teacher-student relationship presumably activates a feeling of safety in teachers, promoting social engagement behavior (Porges, 2001, 2003). This may enable teachers to respond more sensitively and flexibly to challenges such as student aggression. A good teacher-student relationship also likely prevents student misbehavior and may foster prosocial student behavior (Pianta, 2006; Obsuth et al., 2017), which in turn can reduce stress levels among teachers (Aldrup et al., 2018). The perceived better teacher-student relationship may additionally act as a form of social support, contributing to the observed physiological relaxation in teachers.

Reduced HRV can result from elevated stress levels. However, individuals with reduced HRV might also interpret their environment differently. For example, Thayer and Brosschot (2005) demonstrated that people with a lower HRV react to neutral, harmless stimuli as if they were threatening. In contrast, those with higher HRV were better able to adapt their responses to environmental conditions. Consequently, it is plausible that teachers with a lower HRV might view the classroom environment as more hostile even in the absence of threat, increasing the likelihood of engaging in mobilization behavior.

The inverse correlation between HRV and frontal teaching emphasizes the potential stress-inducing nature of this didactic instructional method. Compared to other forms of teaching, frontal teaching is particularly challenging for teachers as they are highly exposed (Wettstein, 2008). They have to impart knowledge to the students and react immediately and adaptively to what is happening in the classroom. Meanwhile, students are relatively inactive and may attempt to go unnoticed or push boundaries (Wettstein, 2008). The reduced HRV observed during frontal teaching may reflect increased vigilance or arousal during these demanding pedagogical practices.

Our study revealed a notable correlation between frontal teaching or perceived teacher-student relationship and HRV, while no significant association with HR was observed. Student aggression was associated with both HRV and HR. It can be assumed that student aggression is related to an activation of the sympathetic nervous system (i.e., increased HR, decreased HRV), as aggression harms social relationships, which is particularly stressful for teachers (Baumeister and Leary, 1995; Epel et al., 2018). In contrast, frontal teaching appears to be a comparatively moderate stressor. This suggests that frontal teaching is related to a release of the vagal brake (i.e., reduced activation of the parasympathetic nervous system) (Porges, 1997).

The different findings regarding the association between RMSSD or SDNN and the risk factors or resources may have occurred since RMSSD and SDNN capture different aspects of HRV, specifically reflecting different branches of the ANS (Shaffer et al., 2014). RMSSD estimates vagally mediated (i.e., parasympathetic) changes in HRV (Shaffer et al., 2014). SDNN is more a measure of the general adaptability of the ANS. It reflects the interplay between the sympathetic and parasympathetic nervous systems (Shaffer et al., 2014), which could influence the different correlations.

### 4.4 Psychological strain

Contrary to our expectations, there were hardly any associations between psychological strain variables and cardiac measures (hypothesis 4). Occupational problems were not associated with teachers’ HR and HRV, and, unexpectedly, there were slightly positive associations between HRV (SDNN) and vital exhaustion.

Generally, findings on the association between chronic stress and HRV have been inconsistent, with hypoactive vagal tone (i.e., reduced HRV) often reported (Keltikangas-Järvinen and Heponiemi, 2004; Thayer and Lane, 2009; Collins and Karasek, 2010). However, increased HRV has been occasionally found with chronic stress (Wekenborg et al., 2019; Matuz et al., 2021; Jiryis et al., 2022). When exhaustion increases, the parasympathetic nervous system can be activated to achieve physiological relaxation. An elevated HRV may help to deal with stress-inducing situations by avoiding them more efficiently (Jiryis et al., 2022). In addition, elevated HRV seems to be associated with more withdrawal from subjectively unmanageable situations (Wekenborg et al., 2019) and with heightened task disengagement (Matuz et al., 2021).

In summary, the physiological response, as indicated by HRV, does not necessarily align with subjective reports of vital exhaustion or occupational stress. Also, other factors, possibly related to the specific dynamics of social interactions in the teaching environment or individual coping mechanisms, may contribute to the observed dissociation. A study by Lupien et al. (2022) suggests that the experience of psychological stress rather reflects poor emotion regulation capacities than physiological stress reactions. It is also conceivable that the relationship between subjective and physiological stress markers is not linear (Lupien et al., 2022).

Thus, physiological stress markers and psychological strain appear to be dissociated. Although commitment and relationship orientation may protect against physiological stress, they come at a price on a psychological level. Relationship work is exhausting.

### 4.5 Limitations and strengths

While our study on teacher stress in the context of social interactions and in light of polyvagal theory provides valuable insights, we must acknowledge certain limitations of our findings. The sample size and the participants’ specific characteristics may constrain our findings’ generalizability. Our study focused on apparently healthy and medication-free teachers, so the results may not be generalizable to all teachers. As the data was collected on one workday and one leisure day for each participant, the teachers’ daily form may also have influenced the results. Given the predominantly cross-sectional nature of the data collection, causality cannot be established. The ambulatory assessment approach employed in our study also has limitations. The reliance on wearable devices for continuous monitoring may introduce measurement errors or technological challenges that could affect the accuracy and reliability of the recorded HR and HRV data. In real-world environments, various factors impact physiological states, such as posture, respiratory depth, physical activity, food and beverage consumption, circadian rhythms, speech, and social situations (Houtveen and de Geus, 2009). Cardiac activity, in particular, is also affected by non-stress-related factors, such as physical activity (Zanstra and Johnston, 2011). The teachers’ different activity levels may also have influenced the cardiac parameters. The absence of experimental control over those factors poses a noteworthy risk of confounding variables. We believe laboratory and field approaches are complementary rather than conflicting strategies; they provide data and perspectives from distinct angles (Wilhelm and Grossman, 2010).

However, since most studies on teacher stress are based on questionnaire surveys or laboratory experiments, the ambulatory assessment approach is also one of the main strengths of our study, as it combines self-report, behavioral observations, and electrophysiological measures of teachers’ everyday working lives. This methodology provides a real-world insight into teachers’ daily experiences (Fahrenberg et al., 2007; Wilhelm and Grossman, 2010; Kockler et al., 2017) and thus allows for a more realistic view of daily stressors and resources. It enables a more thorough understanding of the multifaceted teaching profession. By capturing data in teachers’ naturalistic environments rather than in a controlled laboratory setting, we enhance the ecological validity of our findings. The application of polyvagal theory in the teaching profession is particularly exciting, as this profession involves many complex challenges in social interactions and requires a high degree of flexibility on the part of teachers. By examining HR and HRV in parallel, we gain a comprehensive understanding of ANS dynamics.

### 4.6 Implications

This study emphasizes the great complexity of the teaching profession and contributes to existing literature on the impact of classroom dynamics on teacher well-being. Our study offers various starting points for stress prevention and management for teachers, considering both work and non-work factors. One approach could be strengthening the teacher-student relationship, as this prevents teacher stress. Training teachers in strategies to foster positive connections with students may contribute to students’ academic success (Pianta et al., 2012) and enhance teacher well-being. The findings underscore the importance of pedagogical training addressing student behavior and reevaluating instructional methods to create a supportive and less stressful teaching environment. Reducing teachers’ overall stress levels is crucial to prevent stress from becoming chronic. Equally important is providing support for teachers in developing effective stress management strategies. The implications extend beyond individual classrooms, urging educators, administrators, and policymakers to promote positive school climates and equip teachers with the tools to navigate challenging situations effectively.

## 5 Conclusion

In conclusion, the article’s exploration of teacher stress through the lens of polyvagal theory, using ambulatory assessment of HR and HRV, provides valuable insights into the complex dynamics within educational settings and contributes to our understanding of the factors influencing teacher well-being and health. Positive social relationships are a fundamental human need (Baumeister and Leary, 1995), and the high relevance of social interactions is particularly evident in the teaching profession. Whereas teacher-student interactions can cause stress reactions in teachers (e.g., student aggression), a perceived positive relationship can also contribute to physiological relaxation. It should not be forgotten that building and maintaining positive relationships can be exhausting and come at a psychological cost. Overall, social interactions seem to be crucial for teacher health (Spilt et al., 2011; Aldrup et al., 2018).

## Data Availability

The raw data supporting the conclusions of this article will be made available by the authors, without undue reservation.
